# Diurnal variations in the thickness of the inner bark of tree trunks in relation to xylem water potential and phloem turgor

**DOI:** 10.1002/pei3.10045

**Published:** 2021-05-03

**Authors:** Daniel Epron, Mai Kamakura, Wakana Azuma, Masako Dannoura, Yoshiko Kosugi

**Affiliations:** ^1^ Graduate School of Agriculture Kyoto University Kyoto Japan; ^2^ AgroParisTech INRAE UMR SILVA Université de Lorraine Nancy France; ^3^ Graduate School of Agricultural Science Kobe University Kobe Japan

**Keywords:** *Chamaecyparis obtusa*, dendrometer, gymnosperm, phloem transport, turgor, xylem water potential

## Abstract

The inner bark plays important roles in tree stems, including radial exchange of water with the xylem and translocation of carbohydrates. Both processes affect the water content and the thickness of the inner bark on a diurnal basis. For the first time, we simultaneously measured the diurnal variations in the inner bark thickness of hinoki cypress (*Chamaecyparis obtusa*) by using point dendrometers and those of local xylem potential by using stem psychrometers located next to the dendrometers to determine how these variations were related to each other, to phloem turgor and carbohydrate transport. We also estimated the axial hydrostatic pressure gradient by measuring the osmolality of the sap extracted from the inner bark. The inner bark shrunk during the day and swelled during the night with an amplitude related to day‐to‐day and seasonal variations in climate. The relationship between changes in xylem water potential and inner bark thickness exhibited a hysteresis loop during the day with a median lag of 2 h. A phloem turgor‐related signal can be retrieved from the diurnal variations in the inner bark thickness, which was higher at the upper than at the lower position along the trunk. However, a downward hydrostatic pressure gradient was only observed at dawn, suggesting diurnal variations in the phloem sap flow velocity.

## Graphical abstract

Installation of a two‐point dendrometer at a height of 18.6 m at the base of the crown of a hinoki cypress to measure the diurnal variations in trunk and xylem radius. The inner bark thickness was calculated as the difference between the two radii. One sensor was set on the surface of the inner bark; the second sensor was placed on the xylem surface after 1 cm^2^ of the bark, including the cambium, was removed. Both were attached to a frame fixed to an 18‐cm‐long screw inserted deeply into the trunk.

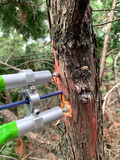

## 1 INTRODUCTION

The inner bark of tree stems is made of a thin layer of living tissue—the secondary phloem—which includes, parenchyma cells, fibres and either sieve cells in gymnosperms or sieve elements in angiosperms. Sieve cells or elements are involved in carbohydrate translocation; fibres provide structural support; and parenchyma cells store water, carbohydrates, and several other compounds such as amino acids, storage proteins, or secondary metabolites (Niklas, 1999; Rosell et al., 2017, 2021; Scholz et al., 2007; Van Bel, 2003).

The whole tree water stores contribute to 10–50% of the daily transpiration of trees during sunny day (probably more on cloudy days), with the water stored in the stem tissues, including the inner bark, which contains more elastic cells than those in the wood, contributing from 5% to 20% (Cermak et al., 2007; Kobayashi & Tanaka, 2001; Köcher et al., 2013; Zweifel & Hasler, 2001; Zweifel et al., 2001). Despite the small contribution of the inner bark to the total stem volume, the contribution of the water stored in the inner bark tissues to the daily water use can be visualised by the diurnal change in the inner bark thickness, which shrinks during the day and swells at night (Chan et al., 2016; Himeno et al., 2017; Lazzarin et al., 2019; Mencuccini et al., 2013; Sevanto et al., 2011; Ueda & Shibata, 2001), with few exceptions (Donnellan et al., 2018; Zweifel et al., 2014). Although most of the change in the inner bark thickness is explained by the changes in the water potential of the xylem (Steppe et al., 2006; Zweifel et al., 2000, 2001), the changes in the amount of solutes in the phloem tissue are thought to contribute to the diurnal variations in the thickness of the inner bark, generating a turgor‐related signal (De Schepper & Steppe, 2010; Mencuccini et al., 2013; Sevanto et al., 2002, 2003; Vandegehuchte et al., 2014).

This turgor‐related signal is expected, because, in addition to storing water, the inner bark also contains the conductive phloem, which is the pathway of carbohydrate translocation between the source organs, mainly the foliage, at least during the growing season, and the sink organs where sugars are used for respiration and biosynthesis (growth and renewal) or are transiently stored. A hydrostatic pressure gradient generated by the difference in osmolality of the phloem sap between the sources and sinks due to loading and unloading of carbohydrates is responsible for the bulk flow through the interconnected sieve cells (Daudet et al., 2002; Knoblauch et al., 2016; Münch, 1930). Hydraulic coupling between two neighbouring tissues—the xylem and phloem—has long been implemented in theoretical models of phloem translocation under the Münch's pressure‐flow hypothesis (Christy & Ferrier, 1973; Daudet et al., 2002; Hölttä et al., 2006, 2009; Thompson & Holbrook, 2003). Although these models are useful in studying the intrinsic properties of the phloem and its interaction with the xylem, their experimental validation has not yet been possible in tall trees.

Nondestructive measurements of diurnal variations in the stem and xylem radius, coupled with models based on Hooke's law, considering the radial exchange of water between the xylem and phloem and the elastic properties of these tissues, allowed the identification of variations in the inner bark thickness that cannot be predicted without taking into account the diurnal changes in the amount of solutes in the phloem tissue, a turgor signal related to phloem loading (Mencuccini et al., 2013; Sevanto et al., 2011). In this study, we recorded diurnal variations in the thickness of the inner bark of mature hinoki cypress trees (*Chamaecyparis obtusa* Sieb. et Zucc.) over several months, by subtracting the xylem radius from the entire trunk radius simultaneously measured using dual‐point dendrometers (Sevanto et al., 2011) at two different heights along the trunk of three trees. For the first time, diurnal variations in inner bark thickness were analysed with those of local xylem water potential measured with stem psychrometers to explore the relationship between inner bark thickness and xylem water potential. The first objective was to clarify how diurnal variations in the inner bark thickness were related to the changes in xylem water potential. Next, we applied the above‐mentioned model of radial water exchange between the phloem and xylem tissues (Mencuccini et al., 2013) to reveal turgor‐related signals in the diurnal changes in the inner bark thickness, which would reveal the dynamics of phloem sugars and its relation to carbohydrate transport. We also measured the axial osmolality gradient of the sap extracted from the inner bark tissues at three occasions and hypothesised the existence of a hydrostatic pressure gradient along the trunk at dawn and in early afternoon, the driving force for long‐distance phloem transport in tall hinoki cypress trees.

## 2 MATERIALS AND METHODS

### 2.1 Experimental site

The experiment was performed in 2019 in a 60‐year‐old hinoki cypress stand in central Japan with sparsely distributed older trees that had been left standing at harvest (Epron et al., 2019). The site is a part of the Kiryu Experimental Watershed (34°58′N, 135°59′E, 250 m above sea level), which belongs to the AsiaFlux network (KEW; Kosugi et al., 2007). The annual air temperature and annual precipitation at the site were 14.2°C and 1707 mm in 2019, respectively, which is typical for this area that is characterized by a warm temperate monsoon climate, with a rainy season in early summer (from mid‐June to the end of July) and potentially tropical cyclones in August and September.

### 2.2 Local climate data

Global radiation (*R*_G_) was measured using a pyranometer (CMP6; Kipp & Zonen B.V., Delft, the Netherlands); air temperature and humidity were measured using a Vaisala sensor (HMP45C; Vaisala) at a height of 28.5 m. Rainfall was measured in a nearby open area using a tipping bucket rain gauge (Ikeda Keiki). All measurements were stored every 10 or 15 min on dataloggers (CR10X; Campbell Scientific Inc.). The vapour pressure deficit was calculated from air temperature and humidity by using R package “plantecophys” (Duursma, 2015).

### 2.3 Trunk radius

Variations in trunk and xylem radius were measured using a two‐point dendrometer (MIJ‐02LM; E.M.J.) attached to a frame fixed to an 18‐cm‐long screw inserted deeply into the trunk heartwood. The distance between the two dendrometers was 6 cm with the screw between them. One dendrometer was set on the surface of the inner bark after the dead outer bark was carefully incised because the hygroscopic properties of the dead outer bark would have contributed to variations in the stem radius (Oberhuber et al., 2020). The second sensor was placed on the xylem surface after 1 cm^2^ of the bark, including the cambium, was removed. The temperature of the frame was measured using a thermocouple, despite the low temperature sensitivity of the device (less than 0.13 µm/°C). Measurement interval was set to 10 min, and the data were stored on a high‐resolution (0.01 mV) datalogger (MIJ‐01; E.M.J.) The resolution of the dendrometer was 2.2 µm/mV (0.022 µm with this datalogger).

Two pairs of dendrometers were installed on three trees (Table 1) at two different heights, one pair at the base of each tree (approximately 1.5 m from the ground, lower position) and the other pair at the base of the crown (upper position), and they were covered with an insulated aluminum foil.

**TABLE 1:** Height (H) and diameter at breast height (DBH) for the three selected trees, with their age and social status; height (*h*) of the lower and upper sensors, and trunk diameters (*d*) at the sensor position; total trunk radius increments at lower and upper positions during the 8 months of measurements

	Tree #1	Tree #2	Tree #3
H (m)/DBH (cm)	22.4/30.6	20.6/18.9	23.5/53.5
Trees’ status and age	Dominant 60 years	Intermediate 60 years	Dominant 90 years
Lower sensors: *h* (m)/*d* (cm)	1.5/29.2	1.7/18.7	1.3/53.5
Upper sensors: *h* (m)/*d* (cm)	17.1/13.7	18.6/7.4	19.5/14.2
Trunk radius increment (mm): lower/upper	0.57/1.71	0.24/1.00	1.98/0.81

### 2.4 Xylem water potential

Xylem water potential (Ψ*_X_*, MPa) was measured at a short distance from point dendrometers by using a stem psychrometer (PSY‐1 Stem Psychrometer; ICT International). A 2 cm^2^ piece of bark (including cambium) was removed, and the sensor was set on the xylem tissue and firmly held with vinyl tape. The remaining part of the exposed xylem was covered with silicon grease. The sensors were insulated with cotton and wrapped with aluminum foil. An aluminum shelter was attached to the stem above the sensor to prevent the entry of rainwater. The data of each sensor were recorded every 10 min by using a microvolt meter (PSY1 Stem Psychrometer Meter; ICT International).

### 2.5 Inner bark osmolality

Two replicates of two inner bark disks with 10 mm diameter were collected at dawn (from 5:00 to 6:30 depending of the time of sunrise) and early afternoon (around 15:00 in summer and around 13:30 in December) on three occasions in August, September, and December at a short distance from the sensors installed on the three trees. The disks were enclosed in plastic bags and immediately stored at −18°C in a portable freezer in the field before being transferred to −30°C in the laboratory. After the disks were thawed in plastic bags, the inner bark samples were weighed fresh. A disk of filter paper was sandwiched between the two discs of the inner bark with the conductive phloem facing the filter, and the samples were pressed to extract the cell sap (Epron et al., 2019). The osmolality of the extracted sap, expressed in mol/kg, was measured using a vapor pressure osmometer (VAPRO 5520; Wescor Inc.) at 25°C in a temperature‐controlled room. The inner bark samples were then dried in an oven, and their dry weight was obtained to calculate the water content of the inner bark.

The hydrostatic pressure (*P*_H_, MPa) in the inner bark tissue, including the turgor pressure and effect of gravity, was estimated by adding the osmotic pressure calculated from the sap osmolality (mean of the two samples) to the xylem water potential that was recorded at the time of sampling:

(1) $$P_{H}=\psi_{X}+C_{S}\times R\times T$$

where *T* is the temperature in Kelvin; *R* the universal gas constant (0.008314 l MPa mol^−1^ K^−1^); *C*_S_ the osmotic concentration, expressed in mol/L, approximated by the osmolality of the sap.

### 2.6 Data analyses

All calculations and analyses were performed using R software, version 3.5.3 (R Core Team, 2019). Unreliable measurements of trunk radius (sensors blocked by resin, resumed cambial growth) and xylem water potential (sensor malfunction) were discarded. Because one had to climb the tree to access the sensors located at the top, we occasionally lost several weeks of data.

The inner bark thickness was calculated as the difference between the radius of the trunk measured on the inner bark and the radius measured on the xylem. The correction for the effect of temperature on the expansion of the frame holding the two sensors is, therefore, cancelled. The inner bark thickness was detrended to remove the seasonal trunk growth (Mencuccini et al., 2013) by using the “stl” function in R (Cleveland et al., 1990). The maximum and minimum inner bark thickness and the times of day when they were observed were extracted. The diurnal amplitude of change in the inner bark thickness was calculated as the difference between the maximum and minimum values.

Linear, polynomial, and exponential relationships between the diurnal amplitude of bark thickness and global radiation, vapour pressure deficit, and air temperature, respectively, were fitted with a linear mixed‐effects model with trees as a random factor. Seasonal apparent Q10 (proportional change in the amplitude for a 10°C variation in air temperature) for each tree was calculated from the model coefficients of the fitted relation between the amplitude and temperature. Cross‐correlations (“ccf” function in R) were used to estimate the lag between diurnal variations of xylem water potential and those of bark thickness.

The model developed by Mencuccini et al., (2013; equation 4) and further used by Chan et al. (2016) and Mencuccini et al. (2017) was used to predict the diurnal variation in the inner bark thickness, assuming that the inner bark tissue is a water reservoir for the xylem and has a constant osmotic content, except that we used the measured xylem water potential (Ψ_X_) instead of the diurnal variation in xylem radius or xylem sap velocity as a proxy for the xylem water potential (Notes S1):

(2) $$\frac{{\text{dTh}}_{\text{IB}}}{dt}=\alpha \left(\beta \Delta \psi_{X}‐\Delta {\text{Th}}_{\text{IB}}\right)+\gamma$$

with adjusted *α*, *β*, and *γ* model parameters. $\frac{{\mathrm{dTh}}_{\mathrm{IB}}}{dt}$ is the change of the inner bark thickness over a time interval d*t*, and ${\Delta \psi }_{X}$ and $\Delta {\mathrm{Th}}_{\mathrm{IB}}$ are the difference in xylem water potential and inner bark thickness relative to the first measurement at midnight. The product *αβ* is the radial hydraulic conductance (see Mencuccini et al., 2013, 2017 for the physiological interpretation of the model parameters). The equation was fitted on the absolute changes in the inner bark thickness over 10‐min interval with a linear model by using generalized least squares (“gls” function in the “nlme” package; Pinheiro et al., 2018) with an autoregressive correlation structure to eliminate the temporal autocorrelation in the residuals due to the dependence of the measurements over time (Mencuccini et al., 2013). The parameters were estimated independently each day and for each tree and position. Occasionally obtained unrealistic adjustments with data collected during rainy days, on days after heavy rain, on very cloudy days, or in late fall were ruled out. The estimated parameters retained were used to predict the changes in the inner bark thickness due to changes in the xylem water potential at a time step of 10 minutes:

(3) $$\Delta {\text{Th}}_{\text{IB}}\left(t+10\right)=\Delta {\text{Th}}_{\text{IB}}\left(t\right)+\alpha \left[\beta \Delta \psi_{X}\left(t\right)‐\Delta {\text{Th}}_{\text{IB}}\left(t\right)\right]$$

The difference between the predicted and observed changes in the inner bark thickness reflects the change due to the variation in the osmotic content of the inner bark, a turgor‐related signal (Mencuccini et al., 2013).

A three‐way analysis of variance was used to estimate the effects of sampling date, sampling time, sampling position, and their interactions on the hydrostatic pressure in the inner bark tissue, whereas a linear mixed‐effects model with trees as a random factor was used for the water content and osmolality of the inner bark because two samples were collected for each tree.

## 3 RESULTS

### 3.1 Annual trunk radial increment and climate

Frequent and abundant rainfall occurred from the end of May (days of year, 140–150) to the end of October (days of year, 300–310) with two periods of more than a week without rain in early August and mid‐September (Figure S1). The minimal daily xylem water potential was never less than −1 MPa and correlated to the maximal daily vapor pressure deficit (VPD; correlation coefficient, 0.44–0.71, depending on the trees and position of the sensors). The maximal daily xylem water potential was always close to 0. The radius of the trunks increased rapidly by 2–20 µm per day on average, depending on the tree until the beginning of August (days of year, 210–220, Figure S1). The total trunk radius increment was lower for the smallest tree (#2) and higher for the largest tree (#3) at the lower position on the trunk; however, at the upper position, the increment was larger for the two 60‐year‐old trees (#1 and #2) than for the oldest one (#3; Table 1).

### 3.2 Amplitude and timing of inner bark shrinkage and swelling

**FIGURE 1:**
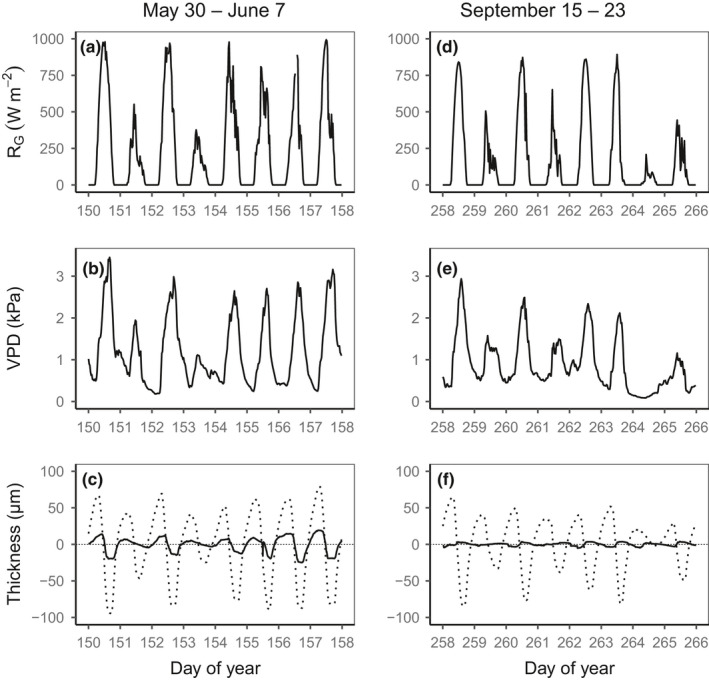
Diurnal courses of (a, d) global radiation (*R*_G_), (b, e) vapor pressure deficit (VPD), and (c, f) detrended inner bark thickness at the upper position (dashed line) and at the lower position (solid line) for tree 1 during nine consecutive days in (a–c) late spring (May 30–June 7) and (d–f) late summer (September 15–23)

**FIGURE 2:**
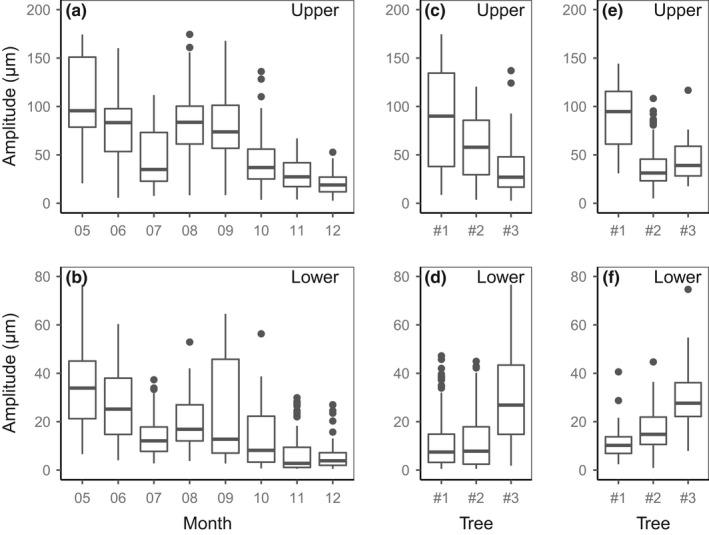
Box plots of the diurnal amplitude of variations in inner bark thickness by month (a) at the upper and (b) at the lower positions, and by tree (c) at the upper and (d) at the lower positions, and box plots of the diurnal amplitude of the turgor signal by trees (e) at the upper and (f) lower positions. The turgor signal is calculated as the difference between the observed and predicted values of the inner bark thickness assuming that the inner bark tissue is a water reservoir for the xylem and has a constant osmotic content (Equation 2). Values for the three trees are combined in (a) and (b), and those of all dates were combined in (c–f). The horizontal thick line is the median; the box, the interquartile range; the whisker, from the largest value no further than 1.5 times the upper quartile to the smallest value at most 1.5 times the lower quartile. Outliers are plotted individually. Note that the extent of the *y*‐axis is different for the upper and lower positions. The number of dates with the amplitude of the turgor signal is lower than with the amplitude of variations in inner bark thickness because unrealistic adjustments were occasionally obtained with data collected during rainy days, on days after heavy rain, on very cloudy days, or in late fall

The inner bark thickness exhibited diurnal variations, decreasing during the day and increasing during the night, with large day‐to‐day fluctuations (Figure 1). Higher amplitudes were observed in May, August, and September than in July (rainy season) and late in the year (amplitude decreased from September to December; Figure 2a,b). At the upper position, over the entire measurement period, the median amplitude was 48 µm with differences between trees (range, 27–90 µm), whereas it was 13 µm at the lower position (range, 8–27 µm). Although trees #1 and #2 had higher amplitudes at the upper position than at the lower position, by a factor of three on average, no such difference was noted for tree #3 (Figure 2c,d).

**FIGURE 3:**
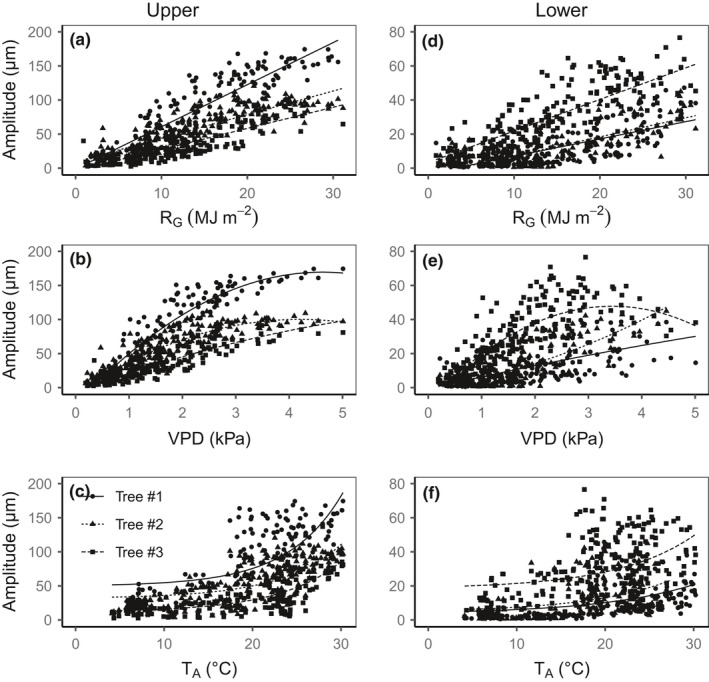
Relationships between the diurnal amplitude of inner bark thickness variations and (a, d) global radiation (*R*_G_, linear trendlines), (b, e) vapor pressure deficit (VPD, polynomial trendlines), and (c, f) air temperature (*T*_A_, exponential trendlines) for tree 1 (circles, solid lines), tree 2 (triangles, dotted lines), and tree 3 (squares, dashed lines). Data are for inner bark thickness at the upper (a–c) and lower positions (d–f). Note that the extent of the *y*‐axis is different for the two positions

The amplitude depended on the climatic conditions, with larger variations on sunny than on cloudy days (Figure 1). On a seasonal basis, the amplitude increased linearly with daily global radiation. The amplitude also increased with the maximal VPD but in a nonlinear fashion; with VPD higher than 3 kPa, the amplitude was no longer related to VPD, especially at the upper position (Figure 3). The amplitude was also exponentially related to the mean air temperature, with a mean apparent Q10 of 2.2 for the upper position with little difference between trees and from 2.1 (tree #3) to 3.0 (trees #1 and #2) for the lower position.

The maximum thicknesses were often recorded a few hours after sunrise, earlier at the upper position (median time, 8:30) than at the lower position (median time, 9:40; Figure 4a). The minimum inner bark thicknesses were also recorded earlier in the evening at the upper position (median time, 16:10) than at the lower position (median time, 19:20; Figure 4b), where it was occasionally recorded late in the night.

**FIGURE 4:**
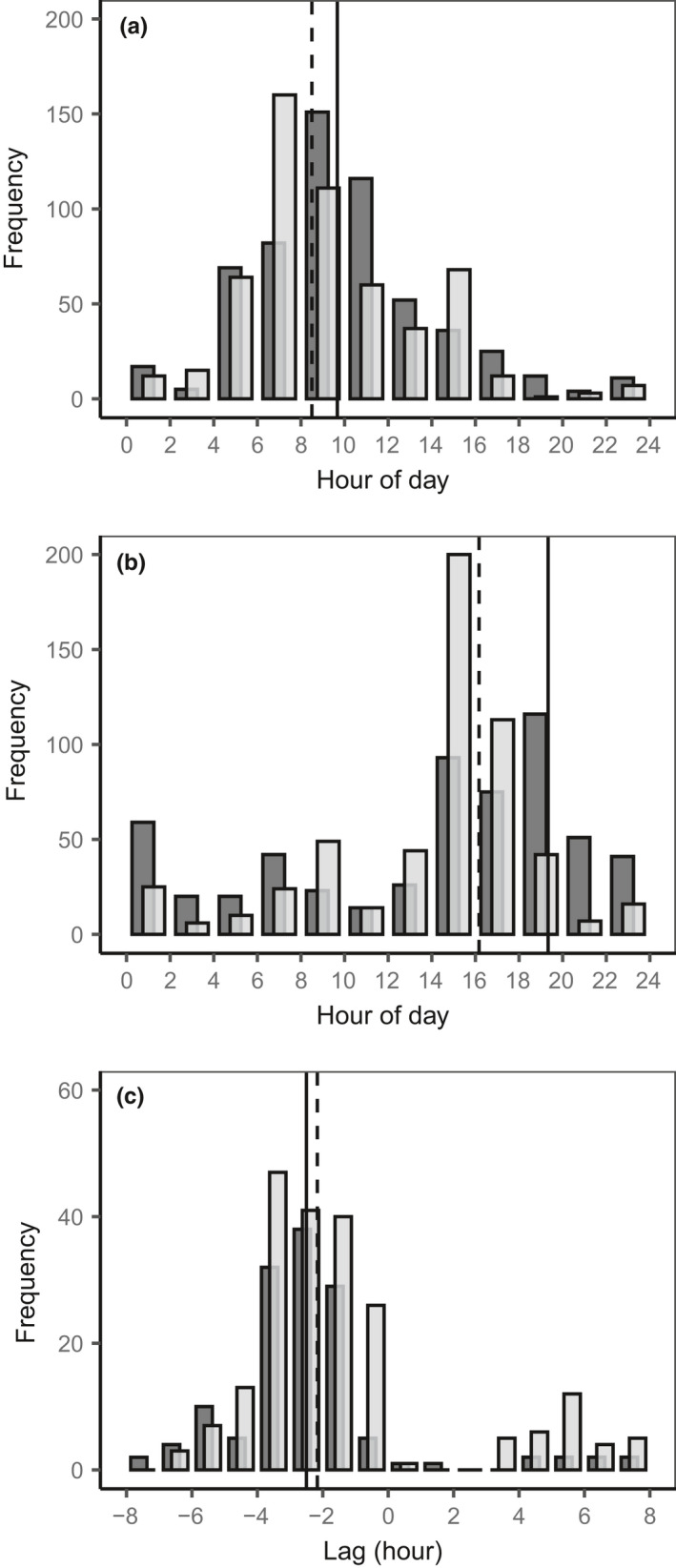
Frequency distribution of the timing of (a) maximum and (b) minimum inner bark thickness, and (c) of the lag of inner bark thickness changes compared to those of xylem water potential, at the upper (light grey) and lower positions (dark grey). All available data between May and December for the three trees are combined. The vertical lines indicate the median at the upper (dashed line) and lower positions (solid line). The calculation of the median of the minimum inner bark thickness takes into account that this value may have occurred after midnight. Negative lag values indicate that the changes in the inner bark thickness lagged behind those of xylem water potential

### 3.3 Diurnal variation of the inner bark thickness and relation with water potential

**FIGURE 5:**
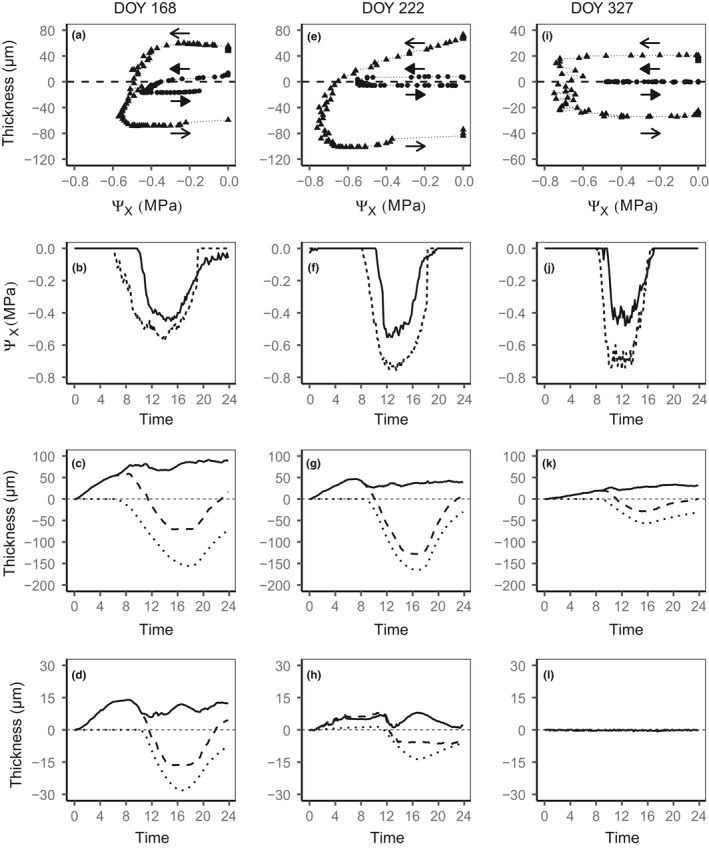
Relationships (a, e, g) between inner bark thickness and xylem water potential (Ψ_X_) from sunrise to sunset at the upper (triangle, open arrows) and lower positions (circles, closed arrows, left arrows for the morning decrease in Ψ_X_ and right arrows for the evening increase in Ψ_X_), and diurnal courses of (b, f, j) xylem water potential (Ψ_X_) at the upper (dotted lines) and lower positions (solid lines), (c, g, k) inner bark thickness (dashed lines), predicted inner bark thickness (dotted lines), and turgor‐related signal (solid line) at the upper and (d, h, l) lower positions on (a, b, c, d) June 17 (day of year, 168) and (e–h) August 10 (day of year, 222) for tree 1 and (i–l) November 23 (day of year, 327) for tree #2. Measured inner bark thickness was adjusted to 0 at the beginning of the day. The turgor signal is calculated as the difference between the observed and predicted values of the inner bark thickness assuming that the inner bark tissue is a water reservoir for the xylem and has a constant osmotic content (Equation 2). Note that the extents of some of the *y*‐axes are smaller in November

Hysteresis was observed between the diurnal variation of xylem water potential and inner bark thickness (Figure 5). At a given water potential, the inner bark was thicker in the morning than in the afternoon, both in the upper and lower positions along the trunk. A steep decrease in the inner bark thickness occurred in a narrow range of xylem water potentials. In most cases, the diurnal decrease in the inner bark thickness lagged behind that of the xylem water potential, with a median value of about 2 h at both positions (Figure 4c).

The predicted changes in the inner bark thickness due to changes in xylem water potential by assuming that the inner bark tissue is a water reservoir for the xylem and has a constant osmotic content. Equation (2) showed a lower diurnal amplitude than the observed changes in the detrended thickness of the inner bark (Figure 5). Over the course of the day, the difference between the observed and predicted values of the inner bark thickness, interpreted as a turgor signal caused by the variation in the osmotic content of the inner bark, increased during the night until mid‐morning, and fluctuated during the day. The diurnal amplitude of the turgor signal was higher in June and August than in November (Figure 5).

When all available data were combined, a higher turgor signal was found at the upper position (median, 38 µm; maximum, 144 µm) than at the lower position (median, 18 µm; maximum, 75 µm), especially for tree #1, which showed higher turgor signal amplitude at the upper position than that of the other trees (Figure 2e,f). No clear relationship was observed between the turgor signal and climatic variables; however, it should be recalled that, in addition to the loss of data due to sensor dysfunctions, rainy days were excluded, and unrealistic adjustments were occasionally obtained and ruled out, especially under very cloudy days or in November and December.

### 3.4 Inner bark osmolality and hydrostatic pressure

Inner bark water content did not differ between the sampling time and sampling position, but was lower in December, especially at the upper position (Table 2). In contrast, the osmolality of the sap extracted from the inner bark was higher in December than in summer and was always higher at the upper position along the trunk than at the lower position. However, the osmolality gradient between the two positions along the trunks was below 0.1 mol/kg in summer. No significant difference in sap osmolality was observed between dawn and early afternoon.

**TABLE 2:** Local climatic conditions: air temperature (*T*_A_) range, maximal VPD, and global radiation (*R*_G_) for the 3 days the samples were collected at dawn and early afternoon at the upper and lower positions of the three trees for measuring inner bark water content and sap osmolality of the inner bark

Date	*T*_A_ (°C)	VPD (kPa)	R_G_ (MJ m^2^)	Time	Position	Inner bark		
						Water content (g/g)	Osmolality (mol/kg)	Hydrostatic pressure (MPa)
Aug 7 (219)	25.5/34.7	2.93	24.5	Dawn	Upper	1.5 (0.2)	0.47 (0.03)	1.16 (0.05)
					Lower	1.3 (0.2)	0.41 (0.05)	1.02 (0.08)
				Early afternoon	Upper	1.5 (0.1)	0.44 (0.06)	0.07 (0.05)
					Lower	1.4 (0.2)	0.37 (0.03)	0.10 (0.05)
Sep 2 (245)	21.6/32.7	2.48	17.9	Dawn	Upper	1.4 (0.2)	0.44 (0.06)	1.05 (0.09)
					Lower	1.4 (0.3)	0.36 (0.08)	0.89 (0.12)
				Early afternoon	Upper	1.4 (0.2)	0.48 (0.01)	0.35 (0.07)
					Lower	1.5 (0.3)	0.41 (0.06)	0.42 (0.04)
Dec 12 (346)	4.1/11.1	0.73	4.7	Dawn	Upper	1.2 (0.1)	0.60 (0.07)	1.50 (0.18)
					Lower	1.4 (0.3)	0.47 (0.04)	1.16 (0.09)
				Early afternoon	Upper	1.2 (0.1)	0.62 (0.13)	1.13 (0.31)
					Lower	1.3 (0.2)	0.48 (0.06)	0.88 (0.21)
Date (*d*)						<0.001	<0.001	<0.001
Time (*t*)						ns	ns	<0.001
Position (*p*)						ns	<0.001	0.009
Interactions						*d* × *p* (0.002)	None	*d* × *t* (<0.001)

Values are the means of two samples on three trees, with standard deviation. The hydrostatic pressure in the inner bark was calculated using xylem water potential at the time of sample collection and the mean osmolality of the two samples (Equation 1). Effects of sampling date (*d*), sampling time (*t*), sampling position (*p*), and their interaction were tested using either a linear mixed‐effects model with trees as random factor for water content and osmolality, or a linear model for the hydrostatic pressure (*p* values are shown).

The estimated hydrostatic pressure was slightly higher at the upper position than at the lower position at dawn and decreased from dawn to early afternoon at both positions, almost zero in August (Table 2). A linear regression between the hydrostatic pressure (*P*_H_) and xylem water potential (Ψ_X_) measured at early afternoon (*P*_H_ = 1.36 + 1.31 Ψ_X_, *R* ^2^ = 0.71) predicted the pressure to be 0 when the xylem water potential dropped below −1.0 MPa. A small hydrostatic pressure gradient between the upper and lower positions was only observed at dawn in summer (less than 0.2 MPa), whereas a higher gradient was observed both at dawn and early afternoon in December (Table 2).

## 4 DISCUSSION

### 4.1 Variations in inner bark thickness

The inner bark of hinoki cypress shrunk during the day and swelled during the night (Figure 1), confirming that, in this species, passive exchange of water occurs between the xylem and phloem tissues (Herzog et al., 1995; Steppe et al., 2006; Zweifel et al., 2000, 2001), driven by the transpiration‐induced diurnal variation of water potential and owing to the elastic phloem tissues for water storage (Rosell et al., 2017; Scholz et al., 2007). A 100 µm variation of inner bark thickness at the upper position, which was the 85th percentile of the observed distribution of the diurnal amplitude when all trees and dates were combined (roughly the mean + SD for a normal distribution), only represents a 4% change in the total water of the inner bark if the change in the volume of the inner bark reflects the change in water content (the volume corresponding to a 100 µm change in the thickness of the inner bark disks collected in summer to measure the water content and osmolality, relative to the average total volume of water in these disks). This small change is consistent with the observed diurnal drop in water potential of between −0.6 and −0.8 MPa (Figure S1 and Figure 5) without considering the change in osmotic pressure in the inner bark, if the modulus of elasticity of the inner bark tissue is between 15 and 20 MPa (Pfautsch et al., 2015). This range of values for the modulus of elasticity was comparable with those reported in previous studies on other species (Génard et al., 2001; Hölttä et al., 2006; Sevanto et al., 2011). In contrast to Norway spruce for which the inner bark exhibited temporal variations in water content (1.4–2.6 g/g), which was larger than predicted from the changes in inner bark thickness (Gall et al., 2002), the inner bark water content (1.4 g/g on average; Table 2) did not change significantly between dawn and early afternoon in hinoki cypress, in agreement with the observed diurnal amplitudes of variations in inner bark thickness.

The pattern of diurnal changes in the inner bark thickness was similar in the upper and lower positions (Figure 1) and similar to that reported for the phloem of hinoki cypress branches (Ueda & Shibata, 2001), suggesting that the same mechanisms operate all along the stem. Despite these similarities, a clear difference in amplitude of diurnal variation was observed between the two positions along the trunk in two of the three trees (Figure 2), probably reflecting the differences in the inner bark thickness (Sevanto et al., 2002). However, a larger diurnal amplitude of radius of the inner bark at the upper position (point measurement) does not necessarily indicate more water exchange compared to that in the lower position because the upper part of the trunk is clearly smaller in diameter than the lower part. Indeed, in terms of water volume, a variation of 100 µm for a stem diameter of 10 cm at the base of the crown is roughly equivalent to a variation of 33 µm for a stem diameter of 30 cm at the base of the trunk. At a given position, the amplitude also showed seasonal and day‐to‐day variation (Figures 1 and 2), in relation to variations in solar radiation, air temperature, and VPD (Figure 3), confirming its dependence on the rate of transpiration (Herzog et al., 1995; Irvine & Grace, 1997; Perämäki et al., 2001). The amplitude was no longer related to the VPD at VPD higher than 3 kPa, possibly due to stomatal closure. The effect of air temperature on amplitude can be indirect, through the effect of VPD, and also direct, through the effect of temperature on the viscosity of water. However, Q10 values of around 2 suggest metabolic controls, which may be related to aquaporin activity (López‐Bernal et al., 2020).

The maximal thickness was mostly observed several hours after sunrise (Figure 4), and the relationship between the changes in xylem water potential and inner bark thickness exhibited a hysteresis loop (Figure 5). In other words, the onset of the shrinkage of the inner bark is delayed in relation to the decrease in water potential. Hysteresis between water potential and trunk diameter variation has commonly been reported (Klepper et al., 1971) and was found to be more important for phloem tissue than for xylem tissues in the branches of hinoki cypress (Ueda & Shibata, 2001). This hysteresis results from the elasticity of tissues and the radial hydraulic conductivity between the phloem and xylem (Génard et al., 2001; Parlange et al., 1975). Diurnal changes in carbohydrate content in the phloem alter the osmotic potential in the phloem cells and may also affect water exchange between the xylem and phloem (Sevanto et al., 2002). A larger lag between the decrease in the inner bark thickness and that in xylem water potential is expected near the source because the sieve cells of the conductive phloem are loaded with carbohydrates, whereas a shorter lag is expected near the sink because of phloem unloading (Sevanto et al., 2003). However, although the maximal and minimal thicknesses of the inner bark were recorded earlier at the upper position than at the lower one, no difference was noted between the two positions in the lag between the change in the inner bark thickness and xylem water potential (Figure 4). The similarity of hysteresis between the two positions is probably due to the fact that the sensors were far enough from the phloem loading that occurred in the small shoots bearing cupressoid leaves. In fact, phloem unloading likely occurred all along the trunk to support active cambial growth of the trunk until August (Figure S1), cell‐wall thickening later on, and respiration of the trunk tissues at all times, which may explain why the lags did not show any clear seasonal variation.

The median values of radial hydraulic conductance that can be derived from the estimated parameters *α* and *β* of the model of radial water exchange between the phloem and xylem tissues (Notes S1, and Mencuccini et al., 2013 for the physiological interpretation of these parameters) were about 8 × 10^−9^ m MPa^−1^ s^−1^ at the upper position and 3 × 10^−9^ m MPa^−1^ s^−1^ at the lower position. These values are slightly lower than those reported for Scots pine (Mencuccini et al., 2013) or broadleaved species (Génard et al., 2001; Sevanto et al., 2011) but are consistent with the rather large lag (a median value of more than 2 h; Figure 4c) between the changes in the inner bark thickness and xylem water potential (Sevanto et al., 2011). The exchange of water between the xylem and the inner bark is thought to be facilitated by the rays of parenchyma cells interconnecting these tissues (Pfautsch et al., 2015). However, despite the evidence of ray of parenchyma in the trunks of hinoki cypress (figure 2A in Epron et al., 2019), their contribution to radial water transport between the inner bark and xylem in gymnosperm species is not yet ascertained (Barnard et al., 2013; Domec et al., 2006; Pfautsch, Hölttä, et al., 2015). If the rays do not facilitate radial water transport in hinoki cypress, it might explain the rather low radial hydraulic conductance.

### 4.2 Relation with phloem osmolality and hydrostatic pressure

Although most of the inner bark tissues act as internal water reserves for transpiration, the thin innermost layer near the cambium—the conductive phloem—has been shown to retain water (De Schepper et al., 2012). Except in the sink tissues, water is supposed to flow from the xylem to the conductive phloem, driven by the variation in the sugar content in the conductive phloem (Hölttä et al., 2006). In contrast to the results obtained for branches of Scots pine (Lazzarin et al., 2019), stems of fast‐growing eucalypts (Zweifel et al., 2014) and the mangrove tree *Avicennia marina* (Donnellan Barraclough et al. 2018; Donnellan et al., 2019), in which day‐time swelling revealed a clear turgor signal, the inner bark of hinoki cypress, like that of other tree species, shrank during the day (Figure 1). However, the model of radial water exchange between the phloem and xylem tissues (Mencuccini et al., 2013) revealed a turgor‐related contribution to diurnal changes in the inner bark thickness in hinoki cypress (Figure 5). The diurnal variation of the turgor‐related signal is known to vary across species, with a clear diurnal pattern with a minimum in the early afternoon noted for some species and less marked variations noted for others (Mencuccini et al., 2013, 2017). In hinoki cypress, the turgor‐related signal increased until mid‐morning and then fluctuated during the day (Figure 5).

The osmolality of the sap extracted from the inner bark ranged from 0.36 to 0.62 mol/kg, which was slightly higher than that noted in spring a few years before at the same site (0.30 mol/kg; Epron et al., 2019), and in the same range as for Scots pine and Norway spruce (Lintunen et al., 2016; Paljakka et al., 2017). Although the measured inner bark osmolalities were not significantly different between dawn and early afternoon (Table 2), the model of radial water exchange between the phloem and xylem tissues predicted diurnal variations in solute content. The median values of the expected diurnal changes in pressure related to the amplitude of the modelled turgor‐related signal were about 0.2 MPa at both positions (Notes S1 and Mencuccini et al., 2013). The model detects changes in the pressure of the inner bark that are only caused by the change in the number of moles of solutes. But after a small time lag due to finite hydraulic conductance between the phloem and xylem, the tissue volume also increases due to the influx of water, which is detected by microdendrometers. This explains why the osmolality of the extracted cell sap remains the same. However, one should be aware that some features of the inner bark are not include in the model of radial water exchange between the phloem and xylem tissues that we used. First, the inner bark is made up of several types of cells (functional sieve cells, nonfunctional sieve cells, and parenchyma cells, among other), which may have different elastic properties and also exhibit elastic anisotropy (Almeras, 2008). Second, the radial conductance between the xylem and phloem could exhibit diurnal variations driven by the activity of aquaporins (Steppe et al., 2012).

The measured inner bark osmolality was nevertheless higher at the upper position than at the lower position. Despite this downward osmolality gradient, a downward hydrostatic pressure gradient was only observed at dawn because of the lower minimum xylem water potential at the upper position than at the lower position during the day (Table 2). As predicted by a coupled xylem–phloem transport model, the decrease in water potential when transpiration is high cancelled the hydrostatic pressure gradient in the phloem (Hölttä et al., 2006). Therefore, the velocity of the phloem sap flow might not remain constant over the course of the day in tall gymnosperms, in contrast to observations based on magnetic resonance imaging performed on small angiosperms (Peuke et al., 2001; Terada et al., 2020; Windt et al., 2006).

The hydrostatic pressure in the trunk is considerably lower than in small shoots bearing cupressoid leaves, at least during the growing season (almost 1 MPa at predawn; Epron et al., 2019). Therefore, most of the axial hydrostatic pressure gradient probably occurs between the collection phloem in the foliage and the top part of the conductive phloem in the branches. A leakage‐retrieval mechanism, as revealed by Minchin and Thorpe (1987) and Epron et al. (2016), for example, or solute relays, as postulated by Lang (1979), might be needed to sustain carbohydrate transport if the observed rather uniform hydrostatic pressure along the trunks is confirmed (Hölttä et al., 2009). The turgor‐related signal, which, as mentioned above, is caused by the change in the number of moles of solutes, may reflect a diurnal influx and outflux of water, coupled to a diurnal conversion of starch to sugar in the inner bark tissue or wood parenchyma, accountable for active osmoregulation allowing local turgor pressure gradients in the phloem.

The low velocity of carbohydrate transport in gymnosperms (Dannoura et al., 2011; Epron et al., 2012) can weaken diurnal fluctuations generated by the production and exportation of photosynthates. With a maximal velocity of carbohydrate transport of 0.2 m/h reported for maritime pine in summer (Dannoura et al., 2011), several hours will be required for photosynthates to reach the upper position on the trunk a few meters away from the foliage, and several days will be required to reach the lower position 20 m below. This may explain why no clear relationships were found between the turgor signal and either climate variables or the canopy photosynthesis derived from the net CO_2_ flux above the canopy measured using the eddy covariance method at this site (data not shown). The inner bark thickness responds in a few hours to the changes in transpiration but requires few days to respond to the changes in photosynthesis and phloem loading. In Scots pine, the turgor signal was related to canopy photosynthesis with a lag of 1 day at the top of the tree and 9 days at the bottom, but the highest correlation coefficients were found with a lag of 10 days at the top and 31 days at the bottom (Mencuccini et al., 2013). Because data were lost either due to sensor malfunctions or because we discarded rainy days and unrealistic adjustments obtained under very cloudy days, we have only a few series of turgor signal that last more than a week and none that lasts more than 10 days. Therefore, testing for the lagged correlation between the turgor signal and either climate variables or canopy photosynthesis was impossible. Sensors located closer to the source leaves, on branches for example (Lazzarin et al., 2019), would have been useful to assess the relationship between the osmotic signal and climate variables or canopy photosynthesis.

The highest osmolality values observed in December may be related to an increase in soluble sugar concentrations during autumn for cold acclimation to tolerate freezing temperature in winter (Lintunen et al., 2016; Pagter et al., 2008; Siminovitch et al., 1953). However, a more pronounced increase in osmolality at the upper position than at the lower position along the trunk can also be the result of a strong influence of air temperature on the velocity of carbohydrate transport in evergreen gymnosperms (Dannoura et al., 2011). Low temperature increases the viscosity of the phloem sap and decreases photosynthesis, and thus phloem loading, cancelling the gradient of hydrostatic pressure between the foliage and the upper part of the trunk.

## 5 CONCLUSION

Diurnal variations in the inner bark thickness in hinoki cypress are mainly driven by the changes in xylem water potential. However, because the observed changes in the inner bark thickness could not be completely predicted by assuming a constant osmotic content in the inner bark, a turgor‐related signal was detected, which is in line with the Münch pressure‐flow hypothesis for long‐distance transport of carbohydrates. The weak axial osmotic gradient in the trunk confirms the role of gravity in phloem transport in hinoki cypress, and potentially the importance of a leakage‐retrieval mechanism. Considering that the hydrostatic pressure gradient decreases during the day, and that the turgor signal was the highest a few hours after sunset, the velocity of the phloem sap flow may exhibit diurnal variations in trees, which has to be addressed in future studies even if it remains a challenge for tall trees in the field.

## CONFLICTS OF INTEREST

The authors declare no conflict of interest.

## Supporting information

Fig S1

Note S1
