# Supplementary material for: Diurnal variations in the thickness of the inner bark of tree trunks in relation to xylem water potential and phloem turgor
Source: Plant Environ Interact. 2021 May 3;2(3):112–24. doi: 10.1002/pei3.10045 (PMC10168075; doi:10.1002/pei3.10045)
Supplement: Supplementary file 1 — Fig S1 [file PEI3-2-112-s001.pdf]

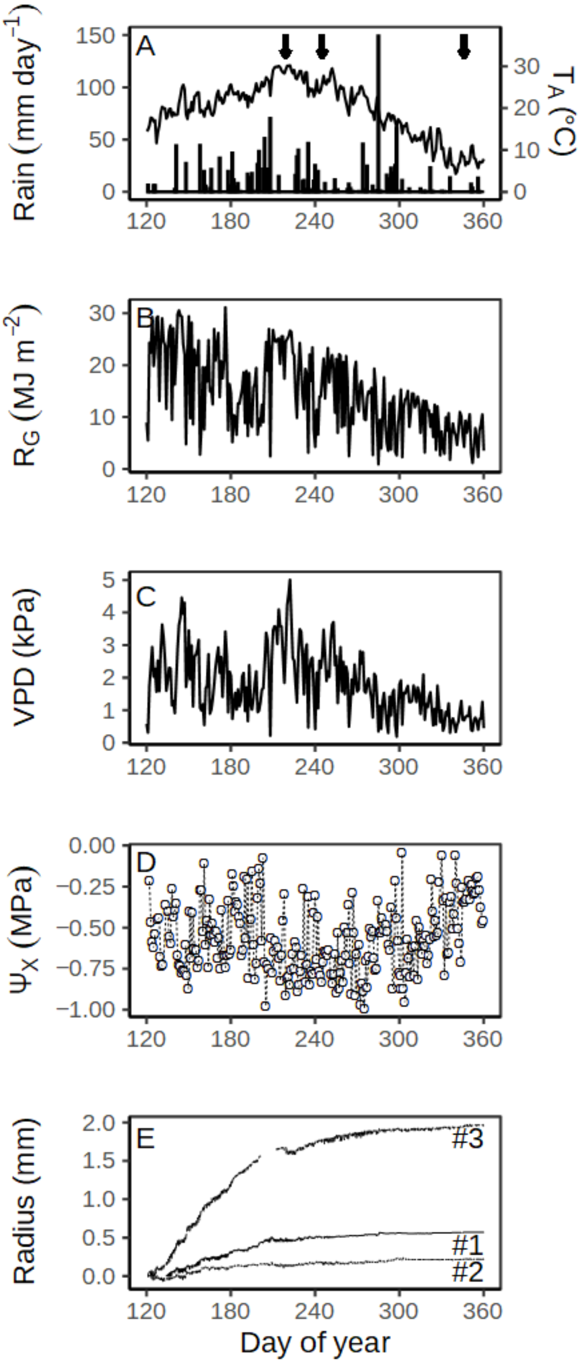

Figure S1: Seasonal courses of (A) mean daily air temperature (T_A) and daily rain, (B) daily global radiation (R_G,), (C) daily maximum vapour pressure deficit (VPD), (D) daily minimum xylem water potential (Ψ_X), and (E) trunk radius (10-min interval). Data in panel D are the mean of the six sensors (three trees and two position, without error bars for clarity), and those in panel E are for the three trees at the lower position on the stem (see Table 1 for the total stem radius increment of each tree at both positions). Missing data are due to either sensor or datalogger malfunction. The thick vertical arrows on panel A point to the three days the samples were collected for measuring the inner bark water content and sap osmolality.
