# Supplementary material for: Diurnal variations in the thickness of the inner bark of tree trunks in relation to xylem water potential and phloem turgor
Source: Plant Environ Interact. 2021 May 3;2(3):112–24. doi: 10.1002/pei3.10045 (PMC10168075; doi:10.1002/pei3.10045)
Supplement: Supplementary file 2 — Note S1 [file PEI3-2-112-s002.pdf]

Supporting information

Notes S1: Model to the diurnal variation in the inner bark thickness

The model has been developed by Mencuccini *et al.* (2013) and further used by Chan *et al.* (2016) and Mencuccini *et al.* (2017). It is based on the Hooke's law, which describe the relation between a small elastic change in the volume and the change in pressure (Irvine & Grace 1997; Perämäki *et al.* 2001; Sevanto, Hölttä & Holbrook 2011). Here, we used the measured xylem water potential instead of the diurnal variation in xylem radius or xylem sap velocity as a proxy for the xylem water potential.

The difference in water potential (Ψ, in MPa) between the xylem (X) and the inner bark (IB), and the radial hydraulic conductance (k, in m^3 MPa^{-1} s^{-1}) determines radial flux of water (J, in m^3 s^{-1}) between the two tissues:

$$J = k \times (\Psi_X - \Psi_{IB}) \qquad [1]$$

The water potential in the inner bark has two components, the hydrostatic pressure (P, turgor and gravity) and the osmotic pressure (Π):

$$\Psi_{IB} = \mathrm{P}_{IB} - \Pi_{IB} \qquad [2]$$

with the osmotic pressure, which depends on the number of moles of solute (n_S), the volume which contains the solutes (V), the temperature (T, in K) and the R the gas constant (R, ideal gas constant, 8.314 J K^{-1} mol^{-1}, with J been equivalent to Pa m^3, or 8.314 10^{-6} m^3 MPa K^{-1} mol^{-1}) if we assume a reflexion coefficient of 1 (perfect semipermeable membranes):

$$\Pi = \frac{\mathrm{n}_S}{V} \times R \times T \qquad [3]$$

The change in the inner bark hydrostatic pressure over time due to the radial flux of water is:

$$\frac{dP_{IB}}{dt} = \frac{E \times J}{V_{IB}^{*}} \qquad [4]$$

with E (in MPa), the elastic modulus of the inner bark tissue in the radial direction and V_{IB}^{*} (in m^3) the volume of the inner bark tissue at a reference pressure (when J = 0, which means $\Psi_X = \Psi_{IB}$). The volume can be decomposed into the thickness of the inner bark at a reference pressure (Th_{IB}^{*}) and the area through which the radial exchange of water between the inner bark and the xylem occurs, which gives:

$$\frac{dP_{IB}}{dt} = \frac{E \times J}{A \times Th_{IB}^{*}} = \frac{E}{A \times Th_{IB}^{*}} \times k \times \left(\Psi_X - (\mathrm{P}_{IB} - \Pi_{IB})\right) \qquad [5]$$

The ratio of the conductance to the area is the area-specific radial hydraulic conductance (L, in m MPa^{-1} s^{-1}) is given by:

$$L = \frac{k}{A} \qquad [6]$$

The variation in inner bark hydrostatic pressure over time is therefore

$$\frac{dP_{IB}}{dt} = \frac{E}{Th_{IB}^*} \times L \times \left(\Psi_X - (\mathrm{P}_{IB} - \Pi_{IB})\right) \qquad [7]$$

or

$$\frac{dP_{IB}}{dt} = \alpha \times \left(\Psi_X - (\mathrm{P}_{IB} - \Pi_{IB})\right) \qquad [8]$$

with

$$\alpha = \frac{E}{Th_{IB}^*} \times L \qquad [9]$$

The elastic change of the inner bark thickness over time is related to the change in hydrostatic pressure in the inner bark:

$$\frac{dTh_{IB}}{dt} = \frac{dP_{IB}}{dt} \times \frac{Th_{IB}^*}{E} \qquad [10]$$

Combining the equations [7] and [10], we obtain:

$$\frac{dTh_{IB}}{dt} = L \times \left(\Psi_X - (\mathrm{P}_{IB} - \Pi_{IB})\right) \qquad [11]$$

In practice, the reference pressure (when J = 0, which means $\Psi_X = \Psi_{IB}$) is unknown. It is therefore useful to refer rather to a reference time (in general the first measurement at midnight). At the reference time:

$$\frac{dTh_{IB}^*}{dt} = L \times \left(\Psi_X^* - (\mathrm{P}_{IB}^* - \Pi_{IB}^*)\right) \qquad [12]$$

$\frac{dTh_{IB}^*}{dt}$ is the change in bark thickness at the reference time due to the imbalance between xylem and phloem water potential at this reference time that will be added to the change in bark thickness. Here, we assume that L will not change over the course of the day.

The change in bark thickness over time becomes:

$$\frac{dTh_{IB}}{dt} = L \times \left(\Delta\Psi_X - (\Delta\mathrm{P}_{IB} - \Delta\Pi_{IB})\right) + \frac{dTh_{IB}^*}{dt} \qquad [13]$$

where $\Delta\Psi_X$, $\Delta\mathrm{P}_{IB}$ and $\Delta\Pi_{IB}$ are the differences relative to the reference time (first measurement at midnight).

If there is no change in inner bark solute content ($\Delta\Pi_{IB} = 0$), then:

$$\frac{dTh_{IB}}{dt} = L \times (\Delta\Psi_X - \Delta P_{IB}) + \frac{dTh_{IB}^*}{dt} \quad [14]$$

According to the Hooke's law:

$$\Delta P_{IB} = \frac{E}{Th_{IB}^*} \times \Delta Th_{IB} \quad [15]$$

with ΔTh_{IB} the difference in inner bark thickness relative to the reference time (first measurement at midnight). Combining [14] and [15]:

$$\frac{dTh_{IB}}{dt} = L \times \left(\Delta\Psi_X - \frac{E}{Th_{IB}^*} \times \Delta Th_{IB}\right) + \frac{dTh_{IB}^*}{dt} \quad [16]$$

which can be rearranged:

$$\frac{dTh_{IB}}{dt} = \frac{E}{Th_{IB}^*} \times L \times \left(\frac{Th_{IB}^*}{E} \times \Delta\Psi_X - \Delta Th_{IB}\right) + \frac{dTh_{IB}^*}{dt} \quad [17]$$

and written more simply as:

$$\frac{dTh_{IB}}{dt} = \alpha \times (\beta \times \Delta\Psi_X - \Delta Th_{IB}) + \gamma \quad [18]$$

with

$$\beta = \frac{Th_{IB}^*}{E} \text{ and } \gamma = \frac{dTh_{IB}^*}{dt} \quad [19,20]$$

The radial hydraulic conductance and the elasticity modulus are:

$$L = \alpha \times \beta \text{ and } E = \frac{Th_{IB}^*}{\beta} \quad [21,22]$$

The amplitude of the diurnal variations in the thickness of the inner bark that is not predicted by the model (by the change in the xylem water potential) can be used to estimate the change in hydrostatic pressure due to the variation in the number of moles of solute ($\Delta P_{\Delta n_s}$), using the Hooke's law:

$$\Delta P_{\Delta n_s} = \frac{E}{Th_{IB}^*} \times \left(\Delta Th_{IB,measured} - \Delta Th_{IB,predicted}\right) \quad [23]$$

$$\Delta P_{\Delta n_s} = \frac{\left(\Delta Th_{IB,measured} - \Delta Th_{IB,predicted}\right)}{\beta} \quad [24]$$

References

Chan T., Hölttä T., Berninger F., Mäkinen H., Nöjd P., Mencuccini M. & Nikinmaa E. (2016) Separating water-potential induced swelling and shrinking from measured radial stem variations reveals a cambial growth and osmotic concentration signal. *Plant, Cell & Environment* **39**, 233–244.

Irvine J. & Grace J. (1997) Continuous measurements of water tensions in the xylem of trees based on the elastic properties of wood. *Planta* **202**, 455–461.

Mencuccini M., Hölttä T., Sevanto S. & Nikinmaa E. (2013) Concurrent measurements of change in the bark and xylem diameters of trees reveal a phloem-generated turgor signal. *New Phytologist* **198**, 1143–1154.

Mencuccini M., Salmon Y., Mitchell P., Hölttä T., Choat B., Meir P., … Pfautsch S. (2017) An empirical method that separates irreversible stem radial growth from bark water content changes in trees: theory and case studies. *Plant, Cell & Environment* **40**, 290–303.

Perämäki M., Nikinmaa E., Sevanto S., Ilvesniemi H., Siivola E., Hari P. & Vesala T. (2001) Tree stem diameter variations and transpiration in Scots pine: an analysis using a dynamic sap flow model. *Tree Physiology* **21**, 889–897.

Sevanto S., Hölttä T. & Holbrook N.M. (2011) Effects of the hydraulic coupling between xylem and phloem on diurnal phloem diameter variation. *Plant, Cell & Environment* **34**, 690–703.
